# Urinary Tract Infections in Hospitalized COVID-19 Patients, What’s Up, Doc?

**DOI:** 10.3390/jcm11071815

**Published:** 2022-03-25

**Authors:** Beatriz Díaz Pollán, Gladys Virginia Guedez López, Paloma María García Clemente, María Jiménez González, Silvia García Bujalance, María Rosa Gómez-Gil Mirá

**Affiliations:** 1 Infectious Disease Unit, Internal Medicine Service, La Paz University Hospital, 28046 Madrid, Spain; 2 Clinical Microbiological Service, La Paz University Hospital, 28046 Madrid, Spain; guedezvirginia069@gmail.com (G.V.G.L.); palomamgarciaclemente@gmail.com (P.M.G.C.); sgbujalance@salud.madrid.org (S.G.B.); mrosa.gomezgil@salud.madrid.org (M.R.G.-G.M.); 3 UCICEC AIDS/Infectious Diseases, IdiPAZ, La Paz University Hospital, 28046 Madrid, Spain; jimenezglezmaria@gmail.com

**Keywords:** COVID-19, SARS-CoV-2, UTI, CAUTI, superinfection, coinfection

## Abstract

The SARS-CoV-2 pandemic might have increased the risks of healthcare-associated infections (HAIs); however, several studies of HAI such as urinary tract infections (UTIs) and catheter-associated urinary tract infections (CAUTIs) have shown contradictory results. The aim of this study is to assess the clinical features of UTIs and bacterial isolates from urine samples of hospitalized COVID-19 patients. We conducted a retrospective observational study including 87 COVID-19 patients with UTIs admitted to our centre. Bacterial UTIs presented were 87: 9 (10.3%) community-acquired UTIs (coinfection group) and 78 (89.6%) hospital-acquired UTIs (superinfection group). In the coinfection group, the most frequent type was non-CAUTI with 5 (55.5%) patients; however, the most frequent UTI in the superinfection group was CAUTI, with 53 (67.9%) patients. The median number of days of hospitalization in coinfected patients was lower than superinfection patients: 13 (IQR 11, 23) vs. 34 days (IQR 23, 47) *p* < 0.006. All UTI patients admitted to ICU, 38 (43.7%), belonged to the superinfection group. The mortality rate was 26.4% (23/87), 22/23 in the superinfection group. The most common microorganisms were *E. coli* 27 (28.4%), *E. faecalis* 25 (26.3%) and *E. faecium* 20 (21.1%). There was an increased incidence of *E. faecalis* and *E. faecium* in UTIs as well as hospital-acquired UTIs. This can be related to urethral catheterization during hospitalization, UCI admissions and the number of days of hospitalization.

## 1. Introduction

The COVID-19 pandemic emerged after the first cases were reported in China in December 2019. As of 6 December 2021, more than 265 million cases of COVID-19 caused by SARS-CoV-2 infection have been reported, with more than 5.2 million deaths, according to WHO data [1]. The risk of these patients for severe COVID-19 and hospitalization is higher in people aged ≥60 years, those living in a nursing home or long-term care facility and those with chronic medical conditions [2,3,4]. Two large cohorts of surveillance have identified that 14% of patients required hospitalization, and 2% were admitted to the intensive care unit (ICU) [5,6]. These data have led to one of the main challenges for the global healthcare systems, the large number of patients requiring hospitalization, notably in the ICU. Some studies have identified low rates of coinfections on admission [7,8], even those studies that have analysed bacterial and fungal infections of the respiratory system [9,10,11,12]. When coinfection (community-acquired, diagnosed within the first 48 h since admission) or superinfection (diagnosis ≥48 h from admission, considered as hospital-acquired) was presented, it seems to be associated with more severe COVID-19 and worse outcomes [13,14], especially in critically ill patients [15].

The fact that hospitalized COVID-19 patients often need a higher level of care, coupled with the collapse of healthcare systems at critical incidence periods (during the SARS-COV-2 pandemic), might have increased the risks of healthcare-associated infections (HAIs) [16,17]. Rates of bloodstream infections (BSIs), central-line–associated bloodstream infections (CLABSIs) or hospital-acquired pneumonias have increased compared to prepandemic HAI incidence studies [14,15,16]. In addition, studies of others such as the rate of urinary tract infections (UTIs) and catheter-associated urinary tract infections (CAUTIs) show contradictory results [13,17]. Moreover, in the published papers of coinfections and superinfections of hospitalized COVID-19 patients, UTIs and CAUTIs have been insufficiently described [7,8,14,15,16,17].

The aim of this study is to assess the clinical features of UTIs and the bacterial isolates from urine samples of hospitalized patients with severe COVID-19 during the first two months of the first wave of maximum incidence in our hospital.

## 2. Materials and Methods

### 2.1. Study Design and Patients

We conducted a retrospective observational study of all patients admitted at a tertiary hospital in Madrid, with a confirmed diagnosis of COVID-19 and significative urine culture from 25 February to 4 May 2020. Our large cohort of COVID-19 patients hospitalized was published [18], and there is one subanalysis about respiratory coinfections published [19]. The study was approved by the Clinical Research Ethics Committee of Hospital Universitario La Paz with the code HULP: PI- PI-4321.3.

### 2.2. Data Collection and Definitions

We included in the study all patients aged 18 years and over with positive RT-PCR for SARS-CoV-2 and significative bacterial urine culture who were hospitalized between 25 February and 4 May 2020. Electronic health records and microbiology laboratory data were used to collect information demographics, risk factors and comorbidities, antibiotic therapy and anti-COVID-19 therapy, microbiological data and outcome variables. Conventional microbiological urine cultures were requested when infection was suspected, not by protocol. We only included in the study UTIs defined as the presence of a bacterial positive urine culture with clinical signs of infection and/or worsening organ failure, according to the Centers for Disease Control (CDC), the Spanish Society of Infectious Diseases and the Clinical and Infectious Diseases Society of America clinical practice Guidelines [20,21,22]. UTI data were reviewed to determine the presence of a true clinical UTI and its source. We divided UTIs into asymptomatic bacteriuria (ASB), symptomatic urinary tract infection noncatheter and CAUTI. BSIs were defined according to the CDC [20] and were only included if patients met the rest of the inclusion criteria. Related bacteraemia was defined when the same microorganism was isolated in blood and urine samples. All UTIs were categorized as urinary tract coinfection (community-acquired UTI) or urinary tract superinfection (hospital-acquired UTI). All patients were treated according to the COVID-19 protocol of our institution and the Spanish Agency of Medicine guidelines [23]. We registered acquired resistance mechanisms to antimicrobials of isolates of urine cultures. Outcome variables were ICU admission, length of hospital stay, hospital discharge, mortality and survival until 30 September 2021.

### 2.3. Laboratory Technique

All patients had a nasopharyngeal swab sample positive RT-PCR for SARS-CoV-2. Urine and blood samples were processed with standard procedures to the protocols of the Hospital’s Clinical Microbiology Service. We collected only bacterial urine isolates of more than >100,000 CFU/mL. Bacterial species were identified by matrix-assisted laser desorption ionization-time of flight mass spectrometry (MALDI-TOF MS−Bruker Daltonics, Bremen, Germany). Antimicrobial susceptibilities were determined by automated broth microdilution assay system (Microscan Walkaway^®^, Beckman Coulter, Brea, CA, USA) and interpreted by means of guidelines of European Committee on Antimicrobial Susceptibility Testing (EUCAST) [24].

### 2.4. Statistical Analysis

The median and IQR were used for the quantitative variables and absolute and relative frequencies for the qualitative variables. For the statistical analysis, first an analysis of the distribution of the variables was performed with the Kolmogorov–Smirnov test. As no variable converged to a normal distribution, the Mann–Whitney U test was used for the comparison by origin of coinfection and the Kruskal–Wallis test for the comparison by type of coinfection. The Chi Square test was also used to compare qualitative variables, and Fisher’s exact test, when necessary. Statistical significance was defined as a *p*-value ≤ 0.05. St Statistical analysis was performed using R software (version 4.1.1, R Core Team (2020), Vienna, Austria).

## 3. Results

We determined that 87 out of 2281 (3.81%) COVID-19 patients admitted to our hospital presented fulfilled inclusion criteria for the study. Clinical characteristics of the patients are shown in Table 1, comparing urinary tract coinfection and urinary tract superinfection of these patients.

### 3.1. Demographic and Epidemiological Data

Forty-eight patients were women (55.2%), and 51 (58.6%) were in the age range of 70–80 years. Among the conditions identified as risks of UTI, urethral catheterization was the most important, followed by urological disease. Urethral catheterization was more frequent in urinary tract superinfections. All UTIs developed in ICU were superinfections.

### 3.2. Type of UTI, Laboratory and Microbiological Characteristics

Community-acquired urinary tract coinfections were uncommon, whereas hospital-acquired urinary tract superinfections were more frequent. Overall, CAUTI was the most common type, although in the coinfection group, non-CAUTI prevailed. Seventy-nine (90.8%) patients had monomicrobial infections vs. three (33.3%) polymicrobial infections, this being a statistically significant association *p* = 0.042. The mean length of stay was significantly longer among patients with urinary tract superinfections. The median days of hospitalization among urinary tract coinfected patients was 13 days (IQR 11–23) and 34 days (IQR 23–47) in hospital-acquired UTI patients, *p* = 0.006. All ICU UTIs were the superinfection type, with almost a month of ICU stay (27 days (IQR 19–37)); 35/38 of them were CAUTI. In the superinfection group, 19 (24.4%) patients presented with bacteraemia (only two outside the ICU), of which 8 (42.1%) were bacteraemia related to a UTI (Table 1).

Details of 95 isolates urine cultures are presented in Table 2. The most common microorganism was *E. coli 27* (28.4%), followed by *Enterococci* (*E. faecalis* 25 (26.3%) and *E. faecium* 20 (21.1%)). A total of 78 (89.6%) patients were treated with targeted antibiotic therapy using their antibiogram. Microorganisms with some acquired resistance mechanisms were registered in 61 patients, the most frequent of which included quinolones resistances, other B-lactams and Gram-negative bacilli extended-spectrum beta-lactamase (ESBL). Gram-negative carbapenemase-producing bacilli type OXA-48 and Gram-negative carbapenemase-producing bacilli type VIM only appeared in UTI superinfections.

### 3.3. Risk Factors for Healthcare-Associated Infection and Outcome

Table 1 describes the main risk factors potentially associated with the development of hospital-acquired infections in COVID-19 patients. The majority of patients on admission were treated with a broad-spectrum antibiotic such as B-lactams (*n* = 63, 72.4%); ceftriaxone was the most frequent (*n* = 49, 56.3%). Other antibiotics used were azithromycin (*n* = 47, 54.0%) or quinolones (*n* = 11, 12.8%). Thirty-two (36.8%) patients received the combination of ceftriaxone and azithromycin. Moreover, 77 (88.5%) patients were given a course of antibiotic treatment for a duration of at least 3 days. In addition, 34 (39.1%) patients received therapy with corticosteroids and/or 18 (20.7%) tocilizumab.

The development of hospital-acquired infections was significantly associated with days of admission, ICU admission and central venous catheter (CVC). A total of 23 (26.4%) patients died during admission; of these, 19 were admitted to the ICU, and 11 presented with bloodstream infection. Four of eight patients with bacteraemia-related infections died, 22 (95.6%) patients died who belonged to the group of urinary tract superinfections and 10 (15.6%) patients died during the follow-up period; all of them belonged to the superinfection group (Table 1).

## 4. Discussion

We studied bacterial UTIs in COVID-19 patients during the first outbreak of the SARS-COV-2 pandemic in Spain. We found a rate 3.81% bacterial urinary tract infection in hospitalized patients (87/2281) where CAUTI was the most frequent type of UTI. There was an increased incidence of *Enterococci* UTI and superinfection type of UTI. Urethral catheter during hospitalization, ICU admissions and numbers of days of hospitalization were higher in the superinfection group.

Infection rates reported by other authors were 1.9 to 9.7% [14,15,25,26]. We have observed that it was particularly difficult to compare UTI rates (3.81% in our study) in COVID-19 patients because the large series in the SARS-COV-2 pandemic did not specifically analyse this type of infection [7,8,12,27] or the UTI definitions used were different according to the author. Thus, in the studies evaluating HAIs, CAUTI was the type of UTI studied, and its incidence was evaluated by different measures: a rate of CAUTIs per 1,000 catheter days, 0.77 [17]; a rate of CAUTIs by number of beds, 1.09 to 2.13 [16]; or a CAUTI rate of 1% by full cohort and 2.4% in the ICU subgroup [28]. UTI-type coinfection in our work is much lower than UTI-type superinfection. We attribute this to the use of urethral catheters and a prolonged stay. Indeed, many or our patients were diagnosed with UTIs in the ICU; they were carriers of a urinary catheter, with a mean stay of 27 days, which predisposes them to greater use of bladder catheters and greater manipulation of the urinary tract. This observation is in agreement with other COVID-19 cohort studies in which the superinfection rates are higher than coinfection rates, mainly in patients admitted to the ICU [14,15,27,29]. The urinary catheter, CVC, ICU admission and a prolonged stay in the ICU are risk factors for healthcare-associated infections that were analysed in our work. These risk factors explain that all UTIs of our ICU patients were nosocomial infections. In addition, 38 patients of the superinfection group stayed in the ICU, 35 were CAUTIs, 37 had CVCs and 17 had a bloodstream infection. The lower rates of coinfections vs. superinfections were in agreement with the rates in the general population [27,29] but opposite to the findings of García-Vidal et al. [14] or those of Gudiol et al. [30], who observed a coinfection rate of UTIs of 27.7% and a superinfection rate of UTIs of 12.7% (in a large cohort of onco-haematological COVID-19 patients).

Women in our cohort presented a higher incidence of bacterial UTIs probably due to the anatomical predisposition. The demographic and clinical features in COVID-19 patients coinfected with UTIs presented older ages and comorbidities as in other studies [7,30]; however, in other work, only comorbidities had been associated [14]. It is difficult to make a comparison between the studies due to the heterogeneity between the populations studied and the scarcity of research on this topic. Notably, urethral catheterization and urological disease were risk factors for hospital-acquired UTI in COVID-19 patients by univariate analysis. In a retrospective study carried out in the USA [17], no increase in CAUTI was found. They attribute this to an augment in antimicrobial use producing the suppression of bacteriuria.

Differences of hospitalized patient populations versus prepandemic hospitalizations could help explain these results as well. To compare the prevalence of UTIs and microbiological aetiology in our patients, we used data from the study on the prevalence of nosocomial infections in Spain of our hospital for 2019 (EPINE-HULP) [31]. Regarding the prevalence of UTIs described in EPINE-HULP 2019, community-acquired UTIs were 19.2% (0.39% in our work) and hospitalized-acquired UTIs were 4.4% (3.42% in our work). The frequency of microorganisms identified shows significant differences between our study and EPINE-HULP 2019: *Enterococci* spp. (*E. faecalis* 25 (26.3%), *E. faecium* 20 (21.1%)), followed by *E. coli* 27 (28.4%) and *Pseudomonas aeruginosa* 7 (7.4%), are the most common microorganisms in our study, while in EPINE-HULP 2019, the most frequent was *Escherichia coli* 11 (39.29%) followed by *Pseudomonas aeruginosa* 2 (7.14%), *Klebsiella* spp. 2 (7.14%) and *Enterococcus faecalis* 1 (3.57%). The increase in UTIs due to *Enterococci* has been described in other works such as Bardi et al. [15], where the most frequent microorganisms were *E. faecalis* and *E. faecium*, but not in the findings of García-Vidal et al. [14], whose main causative agent were Gram-negative bacilli. The increase of *Enterococcus* infections in COVID-19 patients has been analysed in different publications. DeVoe et al. observed that these patients had higher incidence of bloodstream infections (BSIs) due to Enterococcus, but not BSI in general [28]. They did not demonstrate a nosocomial transmission, so the mechanism underlying the increased rates of *Enterococcus* in COVID-19 patients requires further investigation. The use of broad-spectrum antibiotics such as B-lactams, mainly ceftriaxone, may have played a role in changing the microbiota through the selection of less frequent microorganisms such as *Enterococcus*. In addition, antibiotic pressure might select resistance mechanisms such as EBSL or Gram-negative carbapenemase-producing bacilli, as it was observed in our study. Moreover, some types of microorganisms such as Enterococci [32] or *Proteus mirabilis* [33] or certain virulence factors as mrk genes (type3 fimbriae) in *E. coli* and *K. pneumoniae* [34] are associated biofilm infection and might play the role in the CAUTI.

Our findings show an elevated leukocyte count with neutrophilia mainly in patients with urinary tract superinfections, a common condition in the case of bacterial infection. This fact is probably related to the greater number of patients admitted to the ICU and more seriously ill. Biomarkers such as C-reactive protein and procalcitonin were elevated in all cases, although the cytokine storm associated to SARS-COV-2 makes it difficult to interpret these data. In the COVID-19 patient series, they do not appear as risk variables associated with coinfections and superinfections [15,28].

The overall mortality in our study was 26.4%, compared to the initial series of COVID-19 of our hospital, 20.7% [18], and to the bacterial pneumonia coinfections of our hospital, 54.5% [19]. In other series including admitted ICU patients, overall mortality ranged between 9.4 and 38% [14,15,26,30,35].

Limitations of our study include being a retrospective and observational study in a single centre. The diagnostic tests obtained and the procedures performed were conditioned by the daily care without a standardized protocol and a small number of patients limited by period of study. In addition, these results were not corrected for multiple testing, highly increasing the risk of type I errors. Our patients were treated by groups of healthcare workers sometimes not very accustomed to caring for severely ill patients. Furthermore, during the first wave of the pandemic, there were continuous changes in the protocols for the management of COVID-19 patients that may have conditioned prognosis. The impact our local epidemiology and antimicrobial resistance may limit the generalizability of our findings.

## 5. Conclusions

UTIs are infrequent in hospitalized COVID-19 patients; however, when they emerge, they can condition the prognosis. *E. faecalis* or *E. faecium* are the most frequent microorganisms identified, followed by *E. coli*. It is necessary to maintain and develop measures for the prevention of CAUTIs and to assess the true need to maintain the use of urinary catheters and administer antibiotic treatment to avoid infectious complications in this type of patient.

## Figures and Tables

**Table 1:** Main characteristics of population of this study and comparison between urinary tract coinfection and urinary tract superinfection patients.

	All	Urinary Tract Coinfection	Urinary Tract Superinfection	*p*-Value
N	87	9	78	
Gender (%)				1.000
Male	39 (44.8)	4 (44.4)	35 (44.9)	
Female	48 (55.2)	5 (55.6)	43 (55.1)	
Age range (years) (%)				0.913
<50	3 (3.4)	0 (0.0)	3 (3.8)	
50–60	10 (11.5)	1 (11.1)	9 (11.5)	
60–70	23 (26.4)	2 (22.2)	21 (26.9)	
70–80	51 (58.6)	6 (66.7)	45 (57.7)	
Type of UTI (%)				0.080
Asymptomatic bacteriuria	7 (8.0)	2 (22.2)	5 (6.4)	
Symptomatic urinary tract infection noncatheter	24 (27.6)	4 (44.4)	20 (25.6)	
Catheter-associated urinary tract infection	56 (64.4)	3 (33.3)	53 (67.9)	
UTI risk factors (%)				
Urethral catheterization	61 (69.32)	3 (33.3)	58 (74.36)	0.061
Urological disease (anatomic/functionality abnormalities)	17 (19.32)	4 (44.4)	13 (16.67)	0.122
Recurrent urinary tract infections	10 (11.36)	3 (33.3)	7 (8.97)	0.106
Underlying risk factor (%)				
Arterial hypertension	48 (55.2)	9 (100.0)	39 (50.0)	0.012 *
Dyslipidemia	31 (35.6)	6 (66.7)	25 (32.1)	0.092
Cardiovascular disease *	23 (33.3)	3 (42.9)	20 (32.3)	0.888
Diabetes	18 (20.7)	3 (33.3)	15 (19.2)	0.579
Malignancies	9 (10.3)	1 (11.1)	8 (10.3)	1.000
Chronic kidney disease	8 (9.2)	2 (22.2)	6 (7.7)	0.413
Immunosuppressive disease **	7 (8.0)	2 (22.2)	5 (6.4)	0.315
Obesity	6 (6.9)	1 (11.1)	5 (6.4)	1.000
Anti-COVID-19 therapy (%)				
Hydroxychloroquine	81 (93.1)	8 (88.9)	73 (93.6)	1.000
Azithromycin	47 (54.0)	3 (33.3)	44 (56.4)	0.336
Corticosteroids	34 (39.1)	1 (11.1)	33 (42.3)	0.146
Tocilizumab	18 (20.7)	0 (0.0)	18 (23.1)	0.237
Broad-spectrum antibiotics used previously (%)				
B-lactams	63 (72.4)	5 (55.6)	58 (74.4)	0.423
Ceftriaxone	49 (56.3)	4 (44.4)	45 (57.7)	0.686
Linezolid	15 (17.2)	0 (0.0)	15 (19.2)	0.327
Quinolone	11 (12.8)	3 (33.3)	8 (10.4)	0.155
Levofloxacin	9 (10.3)	3 (33.3)	6 (7.7)	0.070
Days of admission (median (IQR))	33.00 (20.50, 45.50)	13.00 (11.00, 23.00)	34.00 (23.00, 47.00)	0.006 *
Days of ICU admission (median (IQR))	27.00 (19.00, 37.00)	0 (0,0)	27.00 (19.00, 37.00)	NA
Risk factors healthcare-associated infections (%)				
Central venous catheter	39 (44.8)	0 (0.0)	39 (50.0)	0.010 *
ICU admission	38 (43.7)	0 (0.0)	38 (48.7)	0.015 *
Bloodstream infection	19 (21.8)	0 (0.0)	19 (24.4)	0.201
Urinary catheter days (median (IQR))	12.00 (8.00, 17.00)	13.50 (8.75, 18.25)	12.00 )8.00, 17.00]	0.879
Bacteraemia-related	8 (42.1)	0 (0)	8 (42.1)	NA
Type of infection by bacterial isolates (%)				0.042 *
Monomicrobial	79 (90.8)	6 (66.7)	73 (93.6)	
Polymicrobial	8 (9.2)	3 (33.3)	5 (6.4)	
Laboratory dates (median (IQR))				
PCR mg/L (median (IQR))	78.10 (25.50, 187.80]	101.60 (53.58, 122.43)	78.10 (25.50, 198.00)	0.940
PCT ng/mL (median (IQR))	0.23 (0.06, 0.59)	0.07 (0.05, 0.13)	0.24 (0.06, 0.60)	0.353
LEUCOS (3.6–10.5) × 10^3^/µL (median (IQR))	9.08 (5.90, 11.83)	6.54 (5.33, 7.05)	9.93 (6.21, 12.34)	0.009 *
LINFO (1.1–4.5) × 10^3^/µL (median (IQR))	0.92 (0.69, 1.40)	1.36 (0.69, 1.57)	0.90 (0.69, 1.30)	0.333
NEUTRO (1.5–7.7) × 10^3^/µL (median (IQR))	6.87 (4.21, 10.21)	4.22 (3.22, 4.25)	7.79 (4.41, 10.69)	0.005 *
PLAQ (150–370) × 10^3^/µL (median (IQR))	268.00 (211.50, 364.50)	268.00 (217.00, 433.00)	264.00 (210.25, 361.75)	0.961
Deaths (%)				0.183
Deaths on admission	23 (26.4)	0 (11.1)	22 (28.2)	
Deaths during follow-up	10 (11.5)	0 (0.0)	10 (12.8)	
Alive	54 (62.1)	8 (88.9)	46 (59.0)	

* Cardiovascular disease includes myocardial infarct, congestive heart failure and peripheral vascular disease. ** Immunosuppressive disease includes HIV infection and patients on chronic treatment with corticosteroids (20 mg/day prednisone or equivalent) or other immunosuppressive treatments.

**Table 2:** Microbiological isolates of our population and comparison between coinfection and superinfection COVID-19 patients.

	All	Urinary Tract Coinfection	Urinary Tract Superinfection	*p*-Value
Isolates Urine Cultures (*n*)	95	12	83	
*Escherichia coli*	27 (28.4)	4 (33.3)	23 (27.7)	
*Enterococcus faecalis*	25 (26.3)	4 (33.3)	21 (25.3)	
*Enterococcus faecium*	20 (21.1)	2 (16.7)	18 (21.7)	
*Pseudomonas aeruginosa*	7 (7.4)	0 (0)	7 (8.4)	
*Klebsiella pneumoniae*	6 (6.3)	1 (8.3)	5 (6.0)	
*Proteus mirabilis*	3 (3.2)	1 (8.3)	2 (2.4)	
*Aerococcus urinae*	1 (1.1)	0 (0)	1 (1.2)	0.519
*Citrobacter freundii*	1 (1.1)	0 (0)	1 (1.2)	
*Citrobacter koseri*	1 (1.1)	0 (0)	1 (1.2)	
*Delftia acidovorans*	1 (1.1)	0 (0)	1 (1.2)	
*Enterobacter cloacae*	1 (1.1)	0 (0)	1 (1.2)	
*Klebsiella aerogenes*	1 (1.1)	0 (0)	1 (1.2)	
*Pseudomonas putida*	1 (1.1)	0 (0)	1 (1.2)	
Acquired resistance mechanisms (%)	61 (64.21)	7 (58.3)	54 (65.1)	0.858
Quinolones	41 (43.2)	6 (50.0)	35 (42.2)	0.841
Other B-lactams	23 (24.2)	4 (33.3)	19 (22.9)	0.668
ESBL *	20 (21.1)	0 (0.0)	20 (24.1)	0.125
Fosfomycin	9 (9.5)	1 (8.3)	8 (9.6)	1.000
Cotrimoxazole	8 (8.4)	0 (0.0)	8 (9.6)	0.570
OXA-48 **	3 (3.2)	0 (0.0)	3 (3.6)	1.000
AmpC ***	2 (2.1)	1 (8.3)	1 (1.2)	0.595
VIM ****	1 (1.1)	0 (0.0)	1 (1.2)	1.000
Aminoglycosides	1 (1.1)	0 (0.0)	1 (1.2)	1.000

* ESBL: Extended-spectrum β-lactamases. ** OXA-48: Carbapenemase-producing bacilli type OXA-48. *** AmpC: AmpC β-lactamases. **** VIM: Verona integron-encoded metallo-β-lactamases.

## Data Availability

The datasets generated during and/or analysed during the current study are not publicly available but are available from the corresponding author on reasonable request.
